# Mental health status of medical students during postgraduate entrance examination

**DOI:** 10.1186/s12888-022-04482-1

**Published:** 2022-12-27

**Authors:** Fajiang Chen, Juanmei Chen, Baoxin Chen, Mohammad Mofatteh, Caijuan Wen, Jack Wellington, Dongchao Gong, Hailing Yang, Zhiyi Zeng, Xiaoyu Miao, Shaoyong Wu, Yimin Chen

**Affiliations:** 1 Graduate School, Guangzhou Medical University, Guangzhou, 511436 China; 2 The Second Clinical College, Guangzhou Medical University, Guangzhou, 511436 China; 3 Faculty of Humanities and Social Sciences, Macao Polytechnic University, Macao, 999078 China; 4 School of Medicine, Dentistry and Biomedical Sciences, Queen’s University Belfast, Belfast, UK; 5 Shunde Polytechnic, Foshan, 528300 China; 6 School of Medicine, Cardiff University, Cardiff, Wales UK; 7 Shunde Polytechnic, Foshan, 528300 China; 8 KingMed School of Laboratory Medicine, Guangzhou Medical University, Guangzhou, China; 9 Department of Research and Education, Foshan Sanshui District People’s Hospital, Foshan, 528100 China; 10 School of Stomatology, Guangzhou Medical University, Guangzhou, 511436 China; 11 Clinical College of Traditional Chinese and Western Medicine, Guangzhou Medical University, Guangzhou, 511436 China; 12 Department of Neurology, Foshan Sanshui District People’s Hospital, Foshan, 528100 China

**Keywords:** Postgraduate entrance examination, Medical students, Mental health, Anxiety, Depression, SCL – 90

## Abstract

**Background:**

The postgraduate entrance examination can be a milestone for many medical students to advance their careers. An increasing number of students are competing for limited postgraduate offers available, and failure to enter postgraduate studies can have adverse mental health consequences. In this paper, we aim to investigate the mental health status of medical students during the postgraduate application entrance examination and to provide a targeted basis for mental health education and psychological counselling.

**Methods:**

Using the Symptom Checklist-90 scale (SCL-90) questionnaire, the mental health status of 613 students who passed two rounds of the Postgraduate Entrance Examination in 2019 to enroll in Guangzhou Medical University in China was evaluated and followed up for retesting 6 months later. We used SPSS 20.0 statistical software for comparative analysis, including One-Sample T-Test, Independent-Samples T-Test, Paired Samples T-Test and Chi-square Test.

**Results:**

Our data showed that 12.10% of students had mental health problems during the postgraduate entrance examination, and it decreased significantly to 4.40% at the 6-month follow-up after the examination period finished (*P* < 0.01). Somatization was the most significant symptom of the students both during and after the postgraduate entrance examination stages. All SCL-90 factors were scored significantly lower both in and after the postgraduate entrance examination stages than the 2008 national college student norm score (*P* < 0.01). Excluding psychiatric factors, all other SCL-90 factors in the postgraduate entrance examination stage scored higher than the graduate stage (*P* < 0.05), and the total score of SCL-90 in female medical students was higher compared to male students (*P* < 0.05).

**Conclusion:**

The postgraduate entrance examination event has a significant negative influence on students’ mental health. The mental health of college and graduate students as an important part of their higher education experience should be systematically studied, and psychological counselling or help should be provided to them throughout their studies, specifically during the examination period. Educating applicants about mental health should be implemented during the postgraduate entrance examination curriculum.

## Introduction

Postgraduate students comprise a large constituent in higher education. In the twenty-first century, global postgraduate education has gradually changed from “elite education” to “mass education” [1, 2]. This transformation is inseparable from the influence exerted by American postgraduate education, progressing into a typical model extending to other countries [3].

**Fig. 1:**
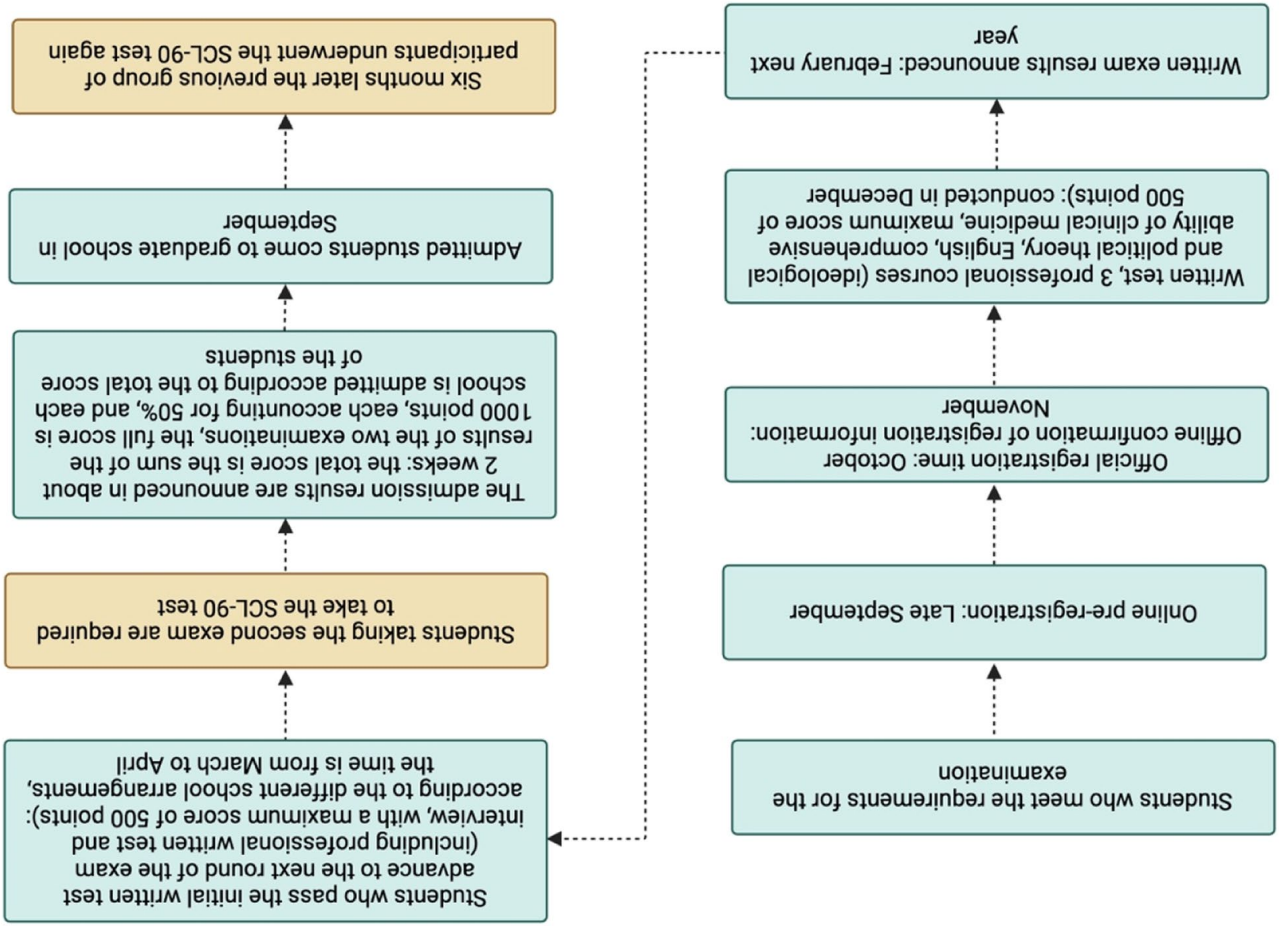
An overview of China’s Postgraduate Entrance Examination Process

In China, the postgraduate entrance examination refers to students with certain conditions to take the national master’s degree examination to pursue a master’s degree. There are two rounds of examinations: the first round is a written test (national unified examination), the second round contains a written test and an interview (organized by each school itself), and only by passing the first round of the written test can students can participate in the second round of written test and interview. After passing both rounds of examinations, applicants will be officially admitted to the Graduate School. Students of all majors can take the postgraduate entrance examination, and the medical major is no exception. Different majors have different examination subjects, and the main examination subjects medical students take are Ideological and Political Theory, English, and Comprehensive Ability of Clinical Medicine. The timing of the first round of postgraduate entrance examinations is in December annually, and the results of the written examination are announced in February of the following year. According to different graduate school arrangements, the second round of written examinations and interviews is generally conducted in March and April. China’s postgraduate entrance examinations have a specific time flow, and its arrangement is shown in Fig. 1.

**Fig. 2:**
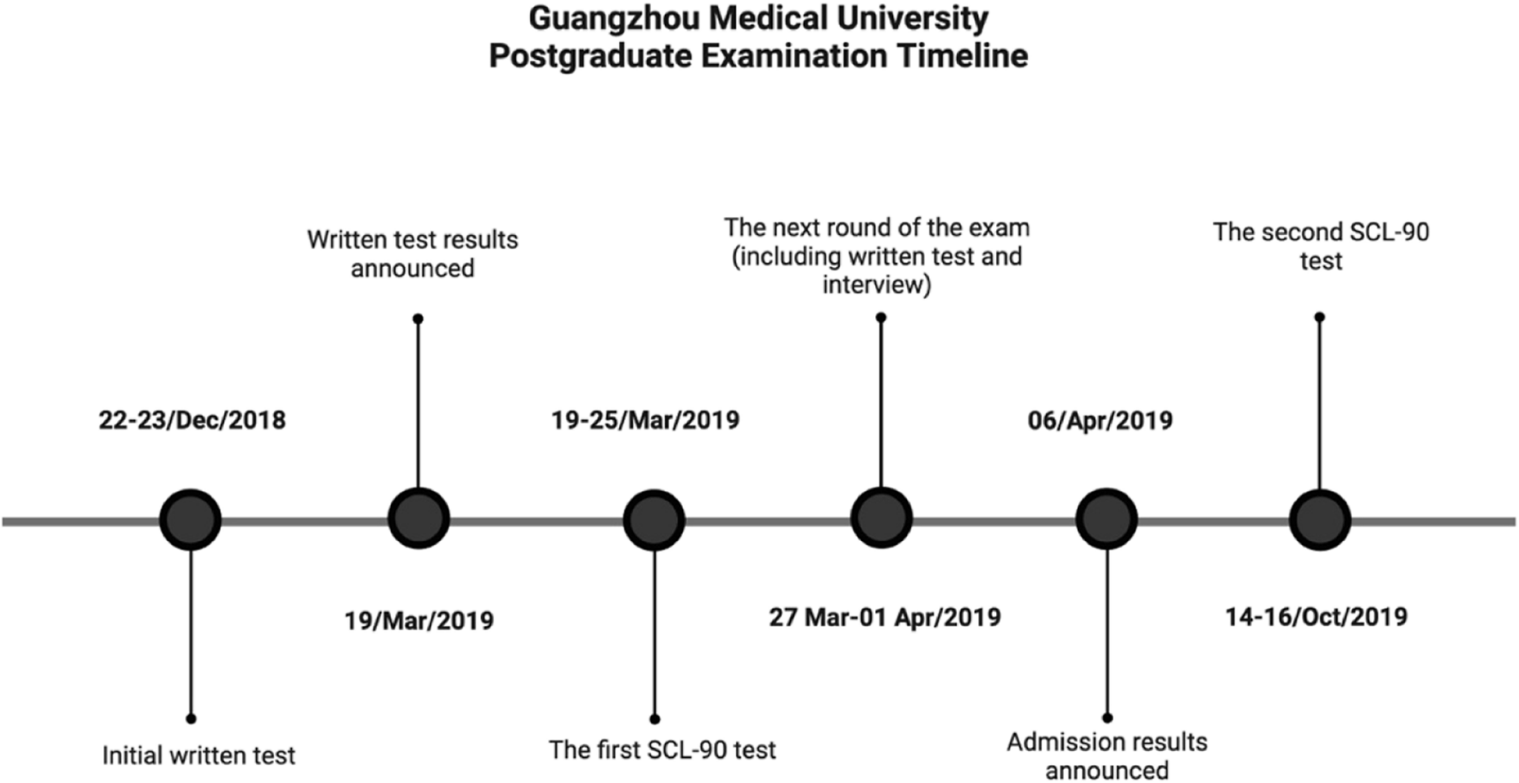
The timeline for the 2019 postgraduate entrance examination for Guangzhou Medical University

Furthermore, the timeline for the 2019 postgraduate entrance examination for Guangzhou Medical University is shown in Fig. 2 as an example.

To increase their competitive employment advantage, the postgraduate entrance examination has become a choice for college students to improve their self-worth [4]. In most societies, access to the best academic institutions is still extremely unequal among individuals from different social backgrounds [5]. Applicants interested in postgraduate applications have increased significantly as Chinese colleges and universities have expanded, and said pressure to access postgraduate higher education to support employability is ever-increasing. The number of applicants for postgraduate entrance examinations in China has increased steadily since 2011, except for a slight decrease in 2014 and 2015. Since 2017, the annual growth rate has been more than 10%. Yang and Li found that 2.9 million of the 8.34 million graduates took the postgraduate entrance exam, but only an estimated 700,000 (24%) were successfully admitted to postgraduate schools. The Chinese Ministry of Education announced that there were 3.41 million applicants who applied for the 2020 postgraduate entrance examination, increasing more than 500,000 over the last year (growth rate of 17%), setting a new high level [6].

Compared to students studying other subjects, medical students face additional challenges caused by clinical practice, continuous assessment, and patient care [7]. Studies supported that medical students are faced with more serious and cumulative anxiety during the preparation period for the exam [8, 9]. Anxiety was the most prevalent symptom among medical students from the Middle East and Asia. The results revealed a 62.17% prevalence of depressive symptoms among medical students during the COVID-19 outbreak [10], due to multiple factors, such as declining interpersonal skills, poor quality of learning experience, lack of work experience, and lost employment opportunities [11]. Numerous studies investigate psychological conditions at the postgraduate level [12–14]. Similarly, the pressure of further education and employment encountered by undergraduates were also significantly associated with anxiety or/and depressive symptoms [15].

Much less attention has been paid to the mental health status of the postgraduate entrance examination population, especially for medical students, who usually encounter significant pressure from arduous subject tasks, more examination subjects, and extensive content than other major students. Therefore, this study aimed to compare and analyze the mental health status of students who participated in the postgraduate entrance examination, exploring the impact of exams on mental health and their influencing factors. Early intervention and improving the mental health of students participating in the postgraduate entrance examination is paramount when ensuring the quality of mental health within postgraduate education.

## Methods

### Participants

The survey was conducted in March 2019 by all students who passed the 2019 China postgraduate entrance examination to enroll at Guangzhou Medical University. A follow-up retest was carried out half a year later. 951 students passed the first writing test, and 45 students did not participate in the first Symptom Checklist 90 scale (SCL-90 test). 906 students took the first SCL-90 test before the postgraduate entrance examination interview, and 293 students were excluded because they were not admitted to the postgraduate school. Overall, 613 valid questionnaires were included. 279 respondents were males, and 334 were females. The mean age of respondents was 21.50 ± 0.60 years old (range 20–23).

### Psychiatric evaluation

The Symptom Checklist 90 scale (SCL-90) questionnaire is a commonly used scale in mental health [16, 17]. SCL-90 is mainly used to describe healthy subjects’ mental health problems or sub-health symptoms, the conscious symptoms, the severity of outpatient or inpatient patients, and the psychological state assessment of various acute and chronic diseases [18–21]. The SCL-90 scale has 90 items, including nine factors of somatization (SOM), obsessive-compulsive (O-C), interpersonal sensitivity (INT), depression (DEP), anxiety (ANX), hostility (HOS), phobic-anxiety (PHO), paranoid ideation (PAR), and psychoticism (PSY) [22].

This study adopted a 5-point Likert scoring method (1 = none, 2 = mild, 3 = moderate, 4 = quite heavy, 5 = severe). The higher the factor score and total average score of the scale, the lower the mental health level. The reference standard total score exceeding 160 points, any factor score exceeding 2 points, or positive items exceeding 43 can be considered a positive result, suggesting potential mental health problems [23, 24].

Professional training investigators instructed students to use the psychological investigation Questionnaire link(SCL-90 scale) in batches to conduct tests within a certain period, using a unified instruction language. After 6 months, the questionnaire retest of the respondents was carried out utilizing an electronic form.

In China, students who take the second round of the postgraduate entrance examination are required to take the SCL-90 test, and after successfully passing the two rounds of the examinations, the school arranges the newly admitted first-year graduate students to take the second SCL-90 test. The second SCL-90 test is approximately 6 months from the first test, and the second test is not mandatory. Most students are willing to take the test, and in this study, all 613 students took the second SCL-90 test with no objections. The importance of mental health is becoming more obvious in China, as reflected by all students participating in the second SCL-90 test. In addition, since medical students have more knowledge about physical and mental health due to their professional training, they are more willing to conduct self-assessments. The participants included were students who took the SCL-90 tests twice, and students who did not participate in the second SCL-90 test were excluded. Therefore, all participating students took the SCL-90 test twice.

### Statistical methods

Statistical analysis was performed using the SPSS 20.0 statistical software. Measurement data were statistically described using mean ± standard deviation and count data using a relative ratio to compare the mental health level of students in the postgraduate entrance examination stage and the graduate student stage with the SCL-902008 national college student norm score [25]. National college student norm reference was published in 2009, and the data of 9941 college students from all 47 colleges were counted in 2008, calculating the mean and standard deviation of the SCL-90 factors as the new norm. We used this norm group as a healthy control college students’ group in a normal social environment for comparison with our college students’ group. Comparative analysis was conducted by statistical methods including One-Sample T-Test, Independent-Samples T-Test, Paired Samples T-Test and Chi-square Test. ***P*** value<0.05 was considered significantly different.

## Results

The influence of postgraduate entrance examination on increased detection rate of mental health problems.

Chi-square Test analysis (Table 1) showed that the positive detection rate of students in the examination stage was significantly higher than the graduate student stage at the 6-month follow-up, and the difference was statistically significant (c^2^ = 23.84, *P* < 0.01).

**Table 1:** Comparison of mental health problems’ positive detection rate of postgraduate entrance examination students and graduate students (10^− 2^)

Item	Positive Number	Positive Detection Rate	c^2^ value	*P* value
Examination Stage	74	12.10	23.84	*P* < 0.001
At 6-month follow-up	27	4.40		

### The influence of postgraduate entrance examination on mental health

The scores before and after the postgraduate entrance examination were compared by Paired T-Test analysis. The results (Table 2) showed that the scores of each factor of SCL-90 in the postgraduate examination stage were significantly higher than those in the graduate student stage, except for the psychoticism factor (*P* < 0.05), indicating that the postgraduate entrance examination has a certain effect on the mental health of medical students.

**Table 2:** Comparison of SCL-90 scores between examination and graduate student stages ($\overline{x}\pm s$, *n* = 613)

Factor	Examination Stage(*n* = 613)	At 6- month follow-up(*n* = 613)	*T* Value	*P* Value
SOM	1.25 ± 0.32	1.19 ± 0.21	5.54	< 0.01
O-C	1.29 ± 0.31	1.26 ± 0.23	3.02	< 0.01
INT	1.19 ± 0.25	1.16 ± 0.19	2.74	0.01
DEP	1.23 ± 0.32	1.17 ± 0.21	5.72	< 0.01
ANX	1.19 ± 0.29	1.12 ± 0.17	6.16	< 0.01
HOS	1.12 ± 0.23	1.10 ± 0.18	2.16	0.03
PHO	1.22 ± 0.33	1.14 ± 0.21	6.05	< 0.01
PAR	1.26 ± 0.32	1.20 ± 0.24	4.30	< 0.01
PSY	1.12 ± 0.26	1.10 ± 0.21	1.66	0.10

*Abbreviations*: *SOM* Somatization, *O-C* Obsessive-compulsive, *INT* Interpersonal sensitivity, *DEP* Depression, *ANX* Anxiety, *HOS* Hostility, *PHO* Phobic-anxiety, *PAR* Paranoid ideation, *PSY* Psychoticism

### The positive detection rate of mental health problems of students in the postgraduate entrance examination stage

The results of this study (Table 3) showed that 74 of the 613 students in the survey had mental health problems, and the positive detection rate of mental health problems was 12.10%. Four SCL-90 factors, somatization, paranoid ideation, obsessive-compulsive and depression had the highest incidence rate.

**Table 3:** Positive detection rate of SCL-90 factors of students in the postgraduate entrance examination stage (10^− 2^)

Factor	Positive Number	Positive Detection Rate
Total	74	12.10
SOM	24	3.92
O-C	17	2.77
INT	9	1.47
DEP	17	2.77
ANX	11	1.79
HOS	3	0.49
PHO	15	2.45
PAR	21	3.43
PSY	3	0.49

### The mental health level of students in the postgraduate entrance examination stage

One-Sample T-Test analysis was used to analyze the scores of SCL-90 between the postgraduate entrance examination students and the 2008 national college student norm. The results (Table 4) show that compared with the 2008 national college student norm, the scores of students in the postgraduate entrance examination stage were lower (*P* < 0.01), indicating that the mental health level of students in the postgraduate entrance examination stage in recent years has improved compared with previous years.

**Table 4:** Comparison of SCL-90 scores between postgraduate entrance examination students ($\overline{x}\pm s$, *n* = 613) and national college student norm (*n* = 9941)

Factor	examination students(*n* = 613)	National college student norm (*n* = 9941)	*T* value	*P* value
SOM	1.25 ± 0.32	1.45 ± 0.49	−15.32	< 0.01
O-C	1.29 ± 0.31	1.98 ± 0.63	− 53.96	< 0.01
INT	1.19 ± 0.25	1.88 ± 0.63	− 68.53	< 0.01
DEP	1.23 ± 0.32	1.74 ± 0.62	− 39.23	< 0.01
ANX	1.19 ± 0.29	1.61 ± 0.55	−36.23	< 0.01
HOS	1.12 ± 0.23	1.61 ± 0.62	−52.12	< 0.01
PHO	1.22 ± 0.33	1.38 ± 0.49	−12.41	< 0.01
PAR	1.26 ± 0.32	1.72 ± 0.62	− 35.61	< 0.01
PSY	1.12 ± 0.26	1.59 ± 0.54	− 44.41	< 0.01

### The decreased detection rate of mental health problems of students at 6- months follow-up after the postgraduate entrance examination

The mental health status of 613 students significantly changed at 6-month follow-up after the end of the postgraduate entrance examination. The results (Table 5) showed that only 27 people had mental health problems, and the positive detection rate was 4.40%. The positive detection rate of the top four mental health problems was consistent with the postgraduate entrance examination stage: somatization, paranoid ideation, obsessive-compulsive, and depression.

**Table 5:** Positive detection rate of SCL-90 at 6- month follow-up after the postgraduate entrance examination (10^− 2^)

Factor	Positive Number	Positive Detection Rate
Total	27	4.40
SOM	3	0.49
O-C	3	0.49
INT	1	0.16
DEP	3	0.49
ANX	1	0.16
HOS	0	0.00
PHO	2	0.33
PAR	3	0.49
PSY	2	0.33

### The mental health level of students at 6- months follow-up after the postgraduate entrance examination

Using the One-Sample T-Test analysis, the mental health level of students at the 6-month follow-up after the postgraduate entrance examination was compared with the 2008 national college student norm (Table 6). The scores of SCL-90 factors after the postgraduate entrance examination stage were also lower than those of the national college student norm (*P* < 0.01), indicating that the mental health level of the surveyed medical students after the postgraduate entrance examination was better than the 2008 national college student norm.

**Table 6:** Comparison of SCL-90 scores at 6- months follow-up after the postgraduate entrance examination ($\overline{x}\pm s$, *n* = 613) and national college student norm score (*n* = 9941)

Factor	At 6-months follow-up(*n* = 613)	National college student norm score(*n* = 9941)	*T* value	*P* value
SOM	1.19 ± 0.21	1.45 ± 0.49	−30.94	< 0.01
O-C	1.26 ± 0.23	1.98 ± 0.63	− 76.78	< 0.01
INT	1.16 ± 0.19	1.88 ± 0.63	− 91.47	< 0.01
DEP	1.17 ± 0.21	1.74 ± 0.62	− 67.94	< 0.01
ANX	1.12 ± 0.17	1.61 ± 0.55	− 71.64	< 0.01
HOS	1.10 ± 0.18	1.61 ± 0.62	− 69.06	< 0.01
PHO	1.14 ± 0.21	1.38 ± 0.49	−28.36	< 0.01
PAR	1.20 ± 0.24	1.72 ± 0.62	−53.33	< 0.01
PSY	1.10 ± 0.21	1.59 ± 0.54	− 57.22	< 0.01

### Mental health status of male and female students in and after the postgraduate entrance examination stage

Independent-Sample T-Test was used to compare the mental health status of male and female students in and after the postgraduate entrance examination stage. The results (Table 7) showed that the total score of female SCL-90 was significantly higher than that of males (*P* < 0.05). Females scored higher than males at interpersonal sensitivity and hostility factors as well (*P* < 0.05). At 6-month follow-up after the postgraduate entrance examination, females scored higher than males in all components except for obsessive-compulsive, depression, hostility, paranoid ideation, and psychoticism factors (*P* < 0.05).

**Table 7:** Comparison of mental health level of males and females in and after the postgraduate entrance examination (*n* = 613)

	Gender	Number	SOM	O-C	INT	DEP	ANX	HOS	PHO	PAR	PSY	Total	The average score for positive items
Postgraduate entrance examination stage	Female	334	1.28 ± 0.32	1.32 ± 0.31	1.19 ± 0.23	1.25 ± 0.32	1.20 ± 0.29	1.14 ± 0.25	1.24 ± 0.33	1.28 ± 0.33	1.13 ± 0.25	112.66 ± 24.48	1.92 ± 0.62
	Male	279	1.22 ± 0.31	1.26 ± 0.31	1.18 ± 0.27	1.21 ± 0.32	1.18 ± 0.28	1.10 ± 0.21	1.19 ± 0.32	1.23 ± 0.31	1.11 ± 0.28	108.61 ± 24.13	1.73 ± 0.87
*T* value			2.7	2.54	0.54	1.38	0.96	2	2.2	2.17	0.95	2.05	3.12
*P* value			0.01	0.01	0.59	0.17	0.34	0.05 (0.046)	0.03	0.03	0.35	0.04	<0.01
At 6-month follow-up	Female	334	1.21 ± 0.22	1.26 ± 0.23	1.17 ± 0.19	1.17 ± 0.21	1.13 ± 0.18	1.10 ± 0.18	1.15 ± 0.22	1.20 ± 0.23	1.10 ± 0.21	107.24 ± 16.49	1.90 ± 0.54
	Male	279	1.17 ± 0.20	1.25 ± 0.24	1.15 ± 0.20	1.16 ± 0.20	1.12 ± 0.15	1.10 ± 0.18	1.13 ± 0.19	1.20 ± 0.25	1.10 ± 0.22	105.47 ± 15.69	1.87 ± 0.63
*T* value			2.38	0.64	1.39	0.53	1.14	0.11	1.43	0.17	0.18	1.35	0.56
*P* value			0.02	0.52	0.16	0.6	0.26	0.92	0.15	0.87	0.86	0.18	0.58

Furthermore, we performed a statistical analysis of the first SCL-90 test results of 293 unadmitted students and compared their scores with 613 admitted students in the first test. There were no statistically significant differences in 8 SCL-90 factors, whereas only depression of unadmitted students was lower than admitted students. But the total score of the two groups was not statistically significant (*P* = 0.324), so we can suggest that students’ psychological states of these two groups were similar.

## Discussion

The postgraduate entrance examination, unlike the ordinary end-of-year final examination, can be considered a life choice for students in some countries, such as China, where entry into postgraduate courses is very competitive. Failure in the postgraduate entrance examination can have negative consequences, such as unemployment. Such stressful life events can have psychological pressure on students [26]. With the substantial increase in the number of postgraduate applications, gaining a place at university becomes more competitive, which can subsequently increase the psychological pressure on students during postgraduate entrance examinations. Stress is a state or feeling achieved when the demand exceeds the limit of an individual’s ability or endurance [27]. While moderate psychological stress can cause arousal in the cerebral cortex, quick thinking, concentration, enhanced memory, and improved learning efficiency, excessive psychological pressure reduces learning efficiency and can lead to serious psychological damage or disease [28]. During the postgraduate examination preparation period, medical students face additional challenges as they must endure the pressure brought by clinical practice, departmental assessment, and patient care [7].

College students are generally in their youth, and their mental endurance, adaptability, and cognitive level can be easily influenced by external factors; therefore, the burden of mental disorders in them was quite high [29]. The presence of external factors based on varying enrollment systems, cultural backgrounds, and difficulty settings for postgraduate entrance examinations causes students to face great pressure, which harms their mental health [30].

However, it is necessary to understand the mindset of current college students and their ability to resist pressure from a spiritual level to truly solve the problem fundamentally, as psychologist Sigmund Freud, “Anxiety arises from a transformation in accumulated tension”. When a person encounters pressure, suppressing emotions can only relieve superficial anxiety but cannot completely solve the deep emotional distress. “The ego is the real source of anxiety”, said Freud [31]. Besides, we can conclude what population characteristics need to be subjected to key psychological intervention in high-pressure situations.

Using the psychometric tool (SCL-90) achieved better mental screening results. It was shown that the positive detection rate of mental health problems in the postgraduate entrance examination stage at a medical school in Guangzhou is 12.10%, which is significantly higher than that of the 6-month follow-up after the postgraduate entrance examination (4.40%). This suggests postgraduate entrance examination has a negative influence on students’ mental health. In addition, whether in or after the postgraduate entrance examination stage, compared with the national college student norm in 2008, students’ SCL-90 score is lower, which is consistent with other relevant studies [32]. However, psychological testing or psychological assessment scale norms should be updated with the times, and the selected data should be representative of time. Considering the impact of factors such as social changes on students taking the postgraduate entrance examination, there are some limitations in choosing the 2008 national college student norm as our standard comparators. When the application of scales lacks time efficiency, it will lead to different levels of performance at different times and in different cultural backgrounds [33]. However, there are few relevant studies on the mental health of college students in China, especially for students taking the postgraduate entrance examination. The 2008 national college student norm is the latest Chinese university student norm we can find so far, and it is the best choice in terms of sample size, data quality and research time. Compared with non-medical students, medical students can better adjust for psychological disorders [34], which may be related to their higher resilience. Medical students have more mental health knowledge, which can help them regulate their poor emotions and psychological states.

The main mental health problems of students in and after the postgraduate entrance examination stage are somatization, obsessive-compulsive, paranoid ideation and depression. In addition to psychoticism factors, compared with students at 6-month follow-up after the postgraduate entrance examination, students during the postgraduate entrance examination stage get higher factor scores, and mental health problems are more serious. Exam preparation is a foremost task for students, during which they have less time for recreational and leisure activities and spending time with friends and family members. This state of social isolation can exacerbate potential mental health problems.

Once the postgraduate entrance examination is over and students successfully enter graduate school, their psychological pressure will be released. In China, the number of graduate applicants is increasing annually, and the limited number of graduate students makes the competition more and more fierce, increasing the psychological pressure on students in the preparation process. After successfully passing two rounds of the exam, the pressure will be partially alleviated or released, and the SCL-90 test score will also decrease accordingly. After 6 months, all the participating students were in their first year of graduate school, which meant they had all passed two rounds of the exam.

We also found that female medical students had a higher SCL-90 total score than males. There was a statistically significant difference in the male and female total scores and 5 of the 10 factors in the first SCL-90 test. A Meta-analysis of studies using the SCL-90 scale to investigate the mental health of Chinese postgraduates also showed that females were more vulnerable than males in experiencing mental health problems [35]. But only 1 of the 10 factors (not the total score) was significantly different in the second SCL-90 test in our study. In addition, we were unable to collect more information on students due to privacy and could not adjust for multiple comparisons (or adjust the statistical tests). Students can experience similar challenges in being admitted to universities. It is still reasonable to explain the difference between male and female students. These findings should be interpreted with caution because of no multiple comparisons or adjust the statistical tests and relatively small sample. Some researchers have suggested that the total scores on the SCL-90 scale may differ in gender, and the calculation of SCL-90 scores and the interpretation of the results may need to be performed separately for different genders. In this study, we calculated SCL-90 scores for females and males according to a consistent standard. The sex difference in mental health during the postgraduate entrance examination should be further studied in the future.

The postgraduate entrance examination has a negative impact on college students’ mental health, and medical students who have special psychological characteristics in the postgraduate entrance examination stage are good at adjusting and controlling their emotions. Targeted mental health education can be implemented to make postgraduate entrance examination students aware of the difficulties or pressures they might encounter during the postgraduate entrance examination process, thereby increasing their awareness and preparing them better for the examination. When they are prepared better mentally for the entrance examination, psychological challenges can be anticipated and dealt with better. In addition, students should receive training on effective psychological adjustment methods, such as time management skills to incorporate reasonable rest and leisure activities while preparing for exams. Females should receive additional support to improve their mental health.

The purpose of our study was to investigate the psychological stress caused by postgraduate entrance examinations on medical students. Comparing the results of the two SCL-90 tests demonstrated that the postgraduate entrance examinations resulted in elevated psychological stress among students. The mental health of college students and graduate students is gaining more attention and is becoming an important component of higher education. Therefore, mental health problems should be studied thoroughly, and psychological counselling services should be provided broadly. Given the importance of mental health in preparing for the examination, students with poor SCL-90 test scores should receive free psychological counselling and guidance. A psychological intervention system should be established in universities to achieve early detection, early diagnosis, and early response to postgraduate students prone to psychological distress to cultivate knowledge, body and psychology-qualified compound talents.

## Limitations and conclusions

Our study has a cross-sectional design, which cannot establish a causal relationship. Furthermore, psychiatric symptoms were self-reported, which can be subject to bias. Despite such limitations, we believe our study can benefit healthcare workers, policymakers, and researchers to better understand the impact of preparing for the postgraduate entrance examination on students’ mental health. It is important to point out that the 2008 norm was relatively outdated, and the new norm should be updated. Psychological testing or psychological assessment scale norms should be updated with the times, the selected data should be representative of the time, and consider the impact of factors such as social changes on students taking the postgraduate entrance examination.

The postgraduate entrance examination event has a significant negative influence on students’ mental health. Therefore, educating applicants about mental health should be implemented during the postgraduate entrance examination.

## Data Availability

The datasets generated and/or analyzed during the current study are not publicly available due to institutional regulations but are available from the corresponding author upon reasonable request.

## Abbreviations

ANX: Anxiety
COVID-19: Coronavirus disease 2019
DEP: Depression
HOS: Hostility
INT: Interpersonal sensitivity
O-C: Obsessive-compulsive
PAR: Paranoid ideation
PHO: Phobic anxiety
PSY: Psychoticism
SCL-90: Symptom checklist-90 scale
SOM: Somatization
